# Comparative Molecular and Microbiologic Diagnosis of Bacterial Endocarditis

**DOI:** 10.3201/eid0912.030229

**Published:** 2003-12

**Authors:** Isabelle Podglajen, Fabienne Bellery, Claire Poyart, Philippe Coudol, Annie Buu-Hoï, Patrick Bruneval, Jean-Luc Mainardi

**Affiliations:** * Hôpital Européen Georges Pompidou, Paris, France; † INSERM E0004, Université Paris VI, Paris, France

**Keywords:** Endocarditis, negative blood culture, molecular diagnosis, PCR, 16S rDNA, *rpoB* and *sodA* genes

## Abstract

Sequencing of 16S rDNA, and of *sodA_int_* and *rpoB_int_* in some cases, was applied to DNA from heart valves of 46 patients (36 with definite and 10 with possible endocarditis). Sequence-based identifications were compared with those obtained with conventional methods. Among the 36 definite cases, 30 had positive blood cultures and 6 had negative cultures. Among the 30 positive cases, sequencing of 16S rDNA permitted identification of species (*18*), genus (*8*), or neither (*4*); *sodA_int_* and *rpoB_int_* sequencing was necessary for species identification in 8 cases. Species identifications were identical in only 61.5%, when conventional techniques and DNA sequencing were used. In five of the six blood culture–negative endocarditis cases, sequencing identified *Bartonella quintana* (*3*), *B. henselae* (*1*), and *Streptococcus gallolyticus* (*1*). Our results demonstrate a clear benefit of molecular identification, particularly in cases of blood culture–negative endocarditis and of possible endocarditis, to confirm or invalidate the diagnosis. Moreover, in 19.4% of the definite cases, the improvement in species identification by sequencing led to improved patient management.

According to the earliest published report on the subject, the prevalence of blood culture-negative endocarditis once ranged from 2.5% to 31% (*1*). In more recent studies, approximately 9% is the reported rate (*2*). One explanation for the improvement in the bacteriologic diagnosis of endocarditis is better knowledge of its clinical symptoms and risk factors, which has encouraged earlier blood culture. Another reason is the improvement in bacterial culture techniques, with prolonged incubation times, presence of carbon dioxide, enriched culture media, and timed subcultures. Thus, the isolation of fastidious microorganisms including *Abiotrophia* (new genus *Granulicatella*) and the HACEK group, has improved dramatically, and organisms frequently missed with the use of earlier blood culture techniques are now recognized. Another improvement is the use of specific serologic tests for certain microorganisms. Such tests, associated with cell cultures, are now recommended for patients with blood culture-negative endocarditis for which *Coxiella burnetii* and *Bartonella spp.* are the suspected causative organisms (*3*–*5*). Despite these improvements, the diagnosis of blood culture–negative endocarditis remains a challenge. The absence of positive culture is most frequently due to previous antimicrobial drug treatment or to bacterial species that are difficult to grow or that remain nonculturable in the laboratory.

To overcome these problems, molecular techniques using broad-range DNA primers for amplification of bacterial 16S rDNA directly from clinical samples and subsequent nucleotide sequencing (*6*,*7*) have been proposed to establish the infectious etiology (*8*–*10*). This approach, combined with sodA_int_, encoding superoxide dismutase (*11*) and rpoB_int_, encoding the β sub-unit of RNA polymerase (*12*) sequencing when 16S rDNA sequences were not sufficiently discriminating, was used here 1) to evaluate the bacterial content of 46 resected heart valves from patients operated on for endocarditis, 2) to compare the results with bacteriologic and histologic findings from heart valves and from preceding blood cultures, and 3) to analyze the data with respect to the clinical background of the patients, including the modified Duke criteria (*13*).

## Patients and Methods

### Patients

From October 2000 to June 2002, all patients operated on for endocarditis at the Hôpital Européen Georges Pompidou, Paris, were classified according to the modified Duke criteria for the diagnosis of infective endocarditis (*13*). We studied a total of 46 cases (26 men and 20 women; average age 55.5 years; range 20–86 years), including 36 clinically definite (31 native valves and 5 prostheses) and 10 clinically possible (5 native valves and 5 prostheses) cases. Among the 36 definite cases, 27 patients had two major criteria, and 9 patients had one major and three minor criteria. All 10 patients classified as having possible endocarditis had one major and one minor criterion. Thirty-two (69.5%) of the 46 patients had been transferred to our hospital for surgery. In these cases, blood cultures and conventional bacterial identification had been performed in other hospitals (23 hospitals, including 3 in foreign countries). Twenty-five patients without endocarditis who were operated on for valve replacement were studied as controls.

### Microbiologic Methods

The excised heart valves were processed under a laminar flow hood. Portions of abnormal valve tissue were ground with a mortar and pestle and cultured on Columbia sheep blood agar, and chocolate agar supplemented with IsoVitaleX (bioMérieux, Marcy l’Etoile, France) (at 37°C aerobically and with 5% CO2 for 10 days), Schaedler sheep blood agar (at 37°C anaerobically for 10 days), brain heart infusion broth, and brain heart infusion broth supplemented with IsoVitaleX (aerobically at 37°C for 30 days). In each case, a valve culture was also performed in an anaerobic blood culture vial (Vital, bioMérieux), which was incubated for 1 month at 37°C. A direct Gram (and Giménez if necessary) stain was performed. Bacteria from isolated colonies were identified according to standard procedures (14). Heart valve samples were stored at – 80°C before DNA extraction. When bacteria were isolated from blood cultures in our hospital, they were identified according to standard procedures (*14*).

### Molecular Methods

#### DNA Extraction

DNA extractions and polymerase chain reactions (PCR) were carried out in separate areas. DNA from heart valve material was extracted (two parallel extractions per valve), according to the manufacturer’s instructions, with the QIAMP Tissue Kit (Qiagen, Courtaboeuf, France). For each batch of extraction, a negative extraction control containing all reagents except heart valve material was included.

### PCR Amplification

For each specimen, DNA in 2 V of crude extract (5 μL and 20 μL) was directly amplified with one or two sets of primers (p13B and p91E or p13B and p515FPL), as described previously for 16S rDNA (*15*), with *d1* and *d2* for *sodA_int_* (*11*) and with CM_7_ and CM_31b_ for *rpoB_int_* (*12*). Reaction mixes (50 μL) were set up with two Taq DNA polymerases (Superpak, Sigma, St. Louis, MO, and Qbiogen, Illkirch, France), according to the specifications of the manufacturers, and 0.2 μM of each appropriate primer. For each batch of six samples, two negative controls were included. PCR was performed in a PTC 200 thermocycler (VWR International, Fontenay-sous-Bois, France) with the following thermal cycling parameters for 16S rDNA: 94°C for 2 min, followed by 35 cycles at 94°C for 30 s, 56°C for 1 min, and 72°C for 1 min, followed by a final extension at 72°C for 10 min. When the PCR result was negative, an amplification of the human β-globin gene was performed as an internal extraction control. Sequence analysis of both strands was carried out on a 3700 DNA analyzer (Applied Biosystems, Courtaboeuf, France). Similarity searches were carried out against GenBank and the Ribosomal Database Project (RDP II, Michigan State University, East Lansing, MI).

### Histologic Analysis

Pieces of formalin-fixed abnormal valve tissue were examined. Paraffin sections were cut and stained with hematoxylin and eosin and Gram (and Giménez if necessary) stains. The histopathologic diagnosis was based on valvular inflammation, vegetation, microorganisms, or other features consistent with endocarditis (*16*).

## Results

Among the 46 cases of endocarditis, 36 were identified before surgery as definite and 10 as possible, according to the modified Duke criteria (*13*).

### Definite Endocarditis

Among the 36 cases of definite endocarditis, blood cultures were positive in 30 and negative in six (Table). After surgery, histopathologic criteria were present in all clinically definite cases.

**Table:** Comparison of results obtained from blood cultures with conventional methods of identification (CMI-BC) and from valves with sequence-based identification (SBI-V)

Positive blood culture (n = 30)					Negative blood culture (n = 6)	
PCR positive (n = 26)				PCR negative (n = 4)^a^	PCR positive (n = 5)	PCR negative n = 1
Species identification						
Concordance (n = 16)	Discordance (n = 10)					
(CMI-BC/SBI-V)	CMI-BC	SBI-V	CMI-BC		SBI-V	
*Streptococcus mutans* *S. sanguis* *S. gallolyticus* (4) *Campylobacter fetus* *Escherichia coli*^b^ *Propionibacterium acnes* *Staphylococcus aureus* (2) *Streptococcus oralis* (2)^c^ *S. mitis* (2)^c^ *S. pneumoniae*^c^	*S. agalactiae* *Gemella spp.* Group C streptococci *S. mitis* (2) *S. sanguis* *S. salivarius* *Haemophilus influenzae* *H. aphrophilus* *Streptococcus spp.*	*Lactobacillus crispatus* *Aerococcus urinae* *Abiotrophia adiacens* *S. sanguis* (2) *S. oralis*^c^ *S. oralis*^c^ *H. aphrophilus* *H. paraphrophilus* *S. gordonii*	*Staphylococcus aureus* *S. epidermidis* *S. caprae* *S. aureus +* *Strep. pneumonia 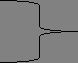 * *+ E. coli*		*S. gallolyticus Bartonella quintana* (3) *B. henselae* d	

^a^In the case of positive blood cultures with *Staphylococcus aureus*, *Escherichia coli,* and *Streptococcus (Strep) pneumoniae*, PCR was positive, but direct sequencing was not interpretable. ^b^Species identification based on sequence analysis of PCR-amplified *rpoB_int_* ^c^Species identification based on sequence analysis of PCR-amplified *sodA_int_* ^d^Three species were identified in the blood culture.

### Blood Culture–Positive Patients

PCR amplification of the bacterial 16S rDNA directly from the resected valves of the 30 patients with positive blood cultures was positive in 26 cases and negative in 4 (Table). The mean duration of antimicrobial drug treatment before surgery was 24.6 days (range 1–75 days) for the PCR-positive group and 34.5 days (range 18–53 days) for the PCR-negative group. In the negative group, three patients had right-side endocarditis stemming from pacemaker infection attributable to *Staphylococcus epidermidis*, *S. caprae,* or an association of *S. aureus*, *Escherichia coli,* and *Streptococcus pneumoniae*, respectively. The fourth patient had endocarditis on the mitral valve attributable to *Staphylococcus aureus*. The direct examination of valve samples after Gram staining showed evidence of bacteria in two cases (*S. aureus* and *S. caprae*); culture was positive in only one, yielding *S. caprae*.

From the 26 valves yielding positive PCR, 5 were culture positive, with the same microorganism as previously identified in the blood cultures (*Escherichia coli*, *S. aureus* [2], *Propionibacterium acnes,* and *Streptococcus mitis*). The mean duration of antimicrobial drug treatment was 8.2 days (range 1–22 days) when the valve culture was positive and 30.2 days (range 5–75 days) when the valve culture was negative. There was full agreement in 16 cases (61.5%) between the bacterial identifications obtained with conventional methods after blood culture and sequence-based identifications with DNA extracted from heart valves (Table). Discrepancies (38.5%, Table) were due to misidentification at the genus level in three cases, misidentification of the species in six cases, and absence of identification to the species level after blood culture in one case. Overall, there were seven cases in which only *sodA_int_* sequence analysis allowed differentiation between *S. mitis*, *S. oralis* and *S. pneumoniae* and one case in which *rpoB* analysis allowed differentiation between *E. coli* and other *Enterobacteriacae*.

### Blood Culture–Negative Patients

In the six cases of negative blood culture (Table), the patients had been treated with antimicrobial drugs before surgery; culture of their valves was also negative. The mean duration of antimicrobial drug treatment was 60 days (range 45–120 days). In five of the six patients, 16S rDNA sequencing permitted bacterial identification from the resected valves. Four of these contained *Bartonella* (three *B. quintana* and one *B. henselae*). Three of the patients with *Bartonella* endocarditis were transferred directly from Africa to our hospital for surgery; primary diagnosis was performed by PCR and confirmed by subsequent serology. In the fourth patient, serologic results were positive before surgery and confirmed by PCR. Serologic testing with a microfluorescence assay showed titers of >1:800 for immunoglobulin G antibodies (*5*). *S. gallolyticus* was identified in the fifth patient, who had been treated with amoxicillin for a suspected urinary tract infection before the blood cultures were taken. In the sixth patient (PCR negative), the histologic results showed a subacute aortic endocarditis with a single epithelioid granuloma, the cause of which remained undetermined.

### Possible Endocarditis

Ten patients were classified before surgery as having possible endocarditis (*13*). In these patients, blood cultures were negative, and PCR did not indicate a microorganism. The histologic analysis of the resected valves also did not indicate any feature of endocarditis, in accordance with the bacteriologic results.

### Control Patients

Twenty-five patients who were operated on for valve replacement but who did not have endocarditis were included as controls. PCR from resected valves and histopathologic signs were negative in all these patients.

## Discussion

The clinical diagnosis of infective endocarditis, particularly in patients who have negative blood culture, were previously treated with antimicrobial drugs, or both, is generally difficult. In this study of 46 cases of definite or possible endocarditis, we used amplification of 16S rDNA extracted from valves and subsequent sequencing to identify the bacterial agent responsible for endocarditis. The results were compared with those obtained with conventional bacteriologic methods of identification after blood culture. When species identifications based on 16S rDNA sequencing were ambiguous, PCR amplification and sequencing of *sodA_int_* or *rpoB_int_* (in one case) were also performed.

When we considered the cases of definite endocarditis with positive blood cultures (Table), there was an agreement of 30% (if one includes the PCR-negative results) between the bacterial identifications obtained after sequencing of 16S rDNA extracted from heart valves and those obtained with conventional techniques after blood culture. There was agreement of 53.3% when the molecular identification included the analysis of *sodA_int_* to differentiate between *S. mitis*, *S. oralis,* and *S. pneumoniae* (*11*) and of *rpoB_int_* to differentiate between *E. coli* and closely related *Enterobacteriacae* (*12*). Combined sequence analysis entailed the refinement of genus to species identification in one case, the revision of species identification in six, and the revision of genus identification in another three. In four cases (13.3%), the PCR result was negative despite positive blood culture (with positive valve culture in one of them) and histologic results that showed features suggestive of endocarditis. This finding was most likely due to the workup of inadequate fractions of the valves (i.e., in some PCR-positive cases, a positive reaction was obtained only with one of the two valve fractions). Multiple fractions should therefore be selected after meticulous macroscopic examination. PCR inhibitors were not likely present since the control reaction with the β-globin gene was positive in all cases. Obviously, no identification can be expected from direct sequencing of PCR products in the presence of multiple bacterial species, as in one case encountered in this study.

If species, as opposed to genus identification, may have only modest consequences on the management of most patients, for some cases the consequences can be substantial. Here, the identification of *Lactobacillus* *crispatus,* instead of *S. agalactiae*, led to the search and treatment of the dental portal of entry and the identification of *Abiotrophia* *adiacens,* instead of group C streptococci; thus in this last case, the antimicrobial drug treatment was prolonged. In five of six cases of negative blood culture, PCR permitted the identification of the responsible microorganism (Table). The identification of *S. gallolyticus* in one patient led to the search for and removal of a precancerous intestinal polyp. In four cases, *Bartonella* species were identified and the antimicrobial drug regimens modified with the introduction of gentamicin (*17*) and prolonged treatment. 16S rDNA PCR is of particular diagnostic value when serology has not been performed and when a serologic test is not available (as in the case of *Tropheryma whippleii*). Moreover, we believe that PCR on resected valves should be performed in patients with positive blood cultures, under two conditions: 1) in the absence of species identifications; or 2) if there is a lack of correlation between the putative microbial identification by conventional microbiologic techniques and the clinical signs and symptoms or course of the endocarditis.

Our study shows that analysis of 16S rDNA extracted from valves is not beneficial only in cases classified as definite endocarditis. It can also serve as a valuable diagnostic tool to confirm or rule out the suspicion of possible endocarditis. The results obtained with this method in the 10 cases we describe were in full agreement with the histologic findings, which did not indicate features of infective endocarditis. All patients for whom negative results were obtained with conventional and molecular methods were secondarily reclassified and were rejected following the Duke’s scheme (*13*). As a therapeutic consequence, the use of unjustified antimicrobial drug treatment was stopped.

We conclude that the diagnosis of bacterial endocarditis may benefit from adding molecular biologic identification to conventional identification after standard cultures. This finding is in agreement with those from a recent study that analyzed a group of patients similar in size and with endocarditis due to a comparable set of infectious agents (*18*) as well as with our recent observation of *C. burnetii*, *Staphylococcus lugdunensis,* and *T. whipplei* in culture-negative valves from endocarditis patients with negative blood cultures. While no false-positive results were obtained with the PCR-based approach in our study, few were false negative. On the other hand, this approach contributes to an improvement in bacterial identification in a substantial number of cases as well as improvement in patient management in approximately 20%.

## References

[1] Cannady PB Jr, Sanford J. Negative blood cultures in infective endocarditis: a review. South Med J. 1976;69:1420–4. 1019634 10.1097/00007611-197611000-00008

[2] Hoen B, Alla F, Selton-Suty C, Béguinot I, Beguinot I, Bouvet A, Changing profile of infective endocarditis. Results of a 1-year survey in France. JAMA. 2002;288:75–81. 10.1001/jama.288.1.75 12090865

[3] Brouqui P, Raoult D. Endocarditis due to rare and fastidious bacteria. Clin Microbiol Rev. 2001;14:177–205. 10.1128/CMR.14.1.177-207.2001 11148009 PMC88969

[4] Maurin M, Raoult D. Q fever. Clin Microbiol Rev. 1999;12:518–53. 10515901 10.1128/cmr.12.4.518 PMC88923

[5] Fournier PE, Mainardi JL, Raoult D. Value of microimmunofluorescence for diagnosis and follow-up of *Bartonella* endocarditis. Clin Diagn Lab Immunol. 2002;9:795–801. 12093675 10.1128/CDLI.9.4.795-801.2002 PMC120030

[6] Carbon P, Ehresmann C, Ehresmann B, Ebel JP. The complete nucleotide sequence of the ribosomal 16-S RNA from *Escherichia coli*. Experimental details and cistron heterogeneities. Eur J Biochem. 1979;100:399–410. 10.1111/j.1432-1033.1979.tb04183.x 389624

[7] Edwards U, Rogall T, Blocker H, Emde M, Böttger EC. Isolation and direct complete nucleotide and determination of entire genes. Characterization of a gene coding for 16S ribosomal RNA. Nucleic Acids Res. 1989;17:7843–53. 10.1093/nar/17.19.7843 2798131 PMC334891

[8] Rantakokko-Jalava K, Nikkari S, Jalava J, Erola E, Skurnik M, Meurman O, Direct amplification of rRNA genes in diagnosis of bacterial infections. J Clin Microbiol. 2000;38:32–9. 10618059 10.1128/jcm.38.1.32-39.2000 PMC86012

[9] Goldenberg D, Künzli A, Vogt P, Zbinden R, Altwegg M. Molecular diagnosis of bacterial endocarditis by broad-range PCR amplification and direct sequencing. J Clin Microbiol. 1997;35:2733–9. 9350723 10.1128/jcm.35.11.2733-2739.1997 PMC230051

[10] Millar B, Moore J, Mallon P, Xu J, Crowe M, Mcclurg R, Molecular diagnosis of infective endocarditis- A new Duke’s criterion. Scand J Infect Dis. 2001;33:673–80. 10.1080/00365540110026764 11669225

[11] Poyart C, Quesne G, Coulon S, Berche P, Trieu-Cuot P. Identification of streptococci to species level by sequencing the gene encoding the manganese-dependent superoxide dismutase. J Clin Microbiol. 1998;36:41–7. 9431917 10.1128/jcm.36.1.41-47.1998 PMC124804

[12] Mollet C, Drancourt M, Raoult D. *RpoB* sequence analysis as a novel basis for bacterial identification. Mol Microbiol. 1997;26:1005–11. 10.1046/j.1365-2958.1997.6382009.x 9426137

[13] Li JS, Sexton DJ, Mick N, Nettles R, Fowler VG Jr, Ryan T, Proposed modifications to the Duke criteria for the diagnosis of infective endocarditis. Clin Infect Dis. 2001;30:633–8. 10.1086/313753 10770721

[14] Murray PL, Baron EJ, Pfaller MA, Tenover FC. Yolken, editors. Manual of clinical microbiology. 7th ed. Washington: American Society for Microbiology; 1999. p. 283–96.

[15] Relman DA, Schmidt TM, MacDermott RP, Falkow S. Identification of the uncultured bacillus of Whipple’s disease. N Engl J Med. 1992;327:293–301. 10.1056/NEJM199207303270501 1377787 PMC13545976

[16] Lepidi H, Durack DT, Raoult D. Diagnostic methods current best practices and guidelines for histologic evaluation in infective endocarditis. Infect Dis Clin North Am. 2002;16:339–61. 10.1016/S0891-5520(02)00005-3 12092476

[17] Raoult D, Fournier PE, Vandenesh F, Mainardi JL, Eykyn SJ, Nash J, Outcome and treatment of *Bartonella* endocarditis. Arch Intern Med. 2003;163:226–30. 10.1001/archinte.163.2.226 12546614

[18] Gauduchon V, Chalabreysse L, Etienne J, Celard M, Benito Y, Lepidi H. Molecular diagnosis of infective endocarditis by PCR amplification and direct sequencing of DNA from valve tissue. J Clin Microbiol. 2003;41:763–6. 10.1128/JCM.41.2.763-766.2003 12574279 PMC149702

